# Workplace and personal predictors of well-being in the broader maternity and neonatal workforce during COVID-19: evidence from a multicentre survey

**DOI:** 10.1186/s12913-026-14588-x

**Published:** 2026-07-28

**Authors:** Alexander J. Lindgren, Leonie Callaway, Kylie Burke, Elizabeth Ryan, Lisa McKeown, Alka Kothari

**Affiliations:** 1 School of Medicine, The University of Queensland, Brisbane, QLD Australia; 2 Metro North Health Service – Redcliffe Hospital, Queensland Health, Redcliffe, QLD Australia; 3 Royal Brisbane and Women’s Hospital (RBWH), Metro North Health, Queensland Health, Herston, QLD Australia; 4 School of Psychology, The University of Queensland, Brisbane, QLD Australia; 5 Metro North Health – Mental Health, Queensland Health, Herston, QLD Australia; 6 Obstetrics and Gynaecology Department, Redcliffe Hospital, Anzac Ave, Redcliffe, QLD 4020 Australia

**Keywords:** Healthcare workforce, Maternity services, Neonatal services, COVID-19, Emotional well-being, Workforce resilience, Health services research, Occupational health, Policy

## Abstract

**Background:**

The COVID-19 pandemic placed unprecedented pressures on maternity and neonatal services, disrupting models of care and amplifying existing stressors for healthcare staff. While much of the literature has focused on doctors and midwives, less is known about the emotional well-being of the broader multidisciplinary maternity and neonatal workforce, including neonatal nurses, sonographers, allied health, and administrative staff. Understanding the collective impact of workplace and personal factors on emotional well-being across this multidisciplinary group is essential for developing effective, inclusive workforce support strategies.

**Methods:**

A cross-sectional survey was conducted among maternity and neonatal staff, including doctors, midwives, nurses, sonographers, allied health, and administrative staff, from one tertiary and two secondary Australian hospitals. Survey responses were analysed using exploratory factor analysis to identify subscales representing key dimensions of emotional well-being, as well as workplace-related and personal factors influencing it. These subscales were then used in multiple hierarchical regression analyses to determine predictors of emotional well-being among staff.

**Results:**

The strongest predictor of emotional well-being was the subscale ‘*Impact of COVID-19 on the Workplace’* (β = -0.345, p < 0.001), encompassing factors such as perceived risk of contracting COVID-19, staff ability to perform duties, and adverse patient outcomes. Other significant predictors included average sleep duration (β = -0.230, p < 0.001), substance use (β = -0.210, p < 0.001), and time spent on outdoor activities (β = -0.130, p < 0.001). The predictive model explained 35.1% of the variance in emotional well-being (R² = 0.351).

**Conclusion:**

Workplace-related and personal factors significantly influenced maternity and neonatal staff well-being during the pandemic. Addressing workplace stress, promoting sleep, supporting healthy coping mechanisms, and encouraging restorative activities may improve staff well-being. These findings can inform the design of targeted workforce support programs and guide service planning strategies to strengthen resilience during future system shocks.

**Trial registration:**

Not applicable.

**Supplementary Information:**

The online version contains supplementary material available at https://doi.org/10.1186/s12913-026-14588-x.

## Background

The onset of the COVID-19 pandemic had a profound impact on the operation of health systems. The pandemic occurred against a backdrop of increasing workforce pressures within healthcare systems globally. Many health systems were already experiencing workforce shortages, increasing workloads, and rising rates of occupational stress and fatigue among healthcare professionals [1–3]. Recent global analyses continue to highlight substantial shortages in the healthcare workforce and their impact on staff workload, well-being, and retention [4, 5]. These pressures were further intensified during the COVID-19 pandemic, as healthcare workers faced prolonged shifts, increased clinical demand, and rapidly evolving clinical protocols [1, 6]. In maternity and neonatal services, such pressures have particular implications because the provision of high-quality care depends not only on technical safety but also on relational and respectful models of care. Pandemic-related service changes, including restrictions on family attendance, infection-control protocols, and rapidly evolving clinical guidance, contributed to constraints on the provision of respectful, woman-centred care and may have increased moral and emotional strain among maternity staff [7].

Within this context, COVID-19 has been shown to significantly impact the mental and physical well-being of healthcare workers globally, with studies reporting staffing challenges, burnout, and impaired patient care [1, 8, 9]. Despite the widespread easing of restrictions, persistent stressors, including periods of increased local COVID-19 transmission, continue to negatively impact healthcare worker performance and mental well-being [10, 11]. Few studies have explored the impact of COVID-19 on maternity and neonatal care providers (MNCPs), and fewer still have examined the broader multidisciplinary maternity and neonatal workforce beyond doctors and midwives [9, 12–14]. The MNCP workforce is particularly vulnerable to public health crises. Baseline stressors affecting MNCPs include exposure to time-critical emergencies, medico-legal risk, shift work, and associated sleep disruption [15]. Staff well-being is not only a determinant of individual health but also closely linked to workforce retention, patient safety, and service quality [2, 16]. Reduced well-being has been associated with higher turnover, absenteeism, and healthcare costs [17, 18], underscoring the importance of addressing these issues as part of health system planning and delivery. To add to these stressors, maternity services underwent significant changes during the COVID-19 pandemic. These changes included restrictions on face-to-face appointments, family attendance, changes or delays in patient care associated with infection control protocols, increased physical burden related to personal protective equipment (PPE) requirements, and impacts on the ability to provide woman-centred care [7, 19, 20].

This study aimed to investigate workplace and lifestyle factors associated with emotional well-being in the broader maternity and neonatal workforce during the COVID-19 pandemic. By identifying and examining the relative contributions of potentially modifiable factors, the findings may help inform workforce support strategies and service planning during periods of major health system disruption.

## Methods

### Study design, settings and participants

A multi-site cross-sectional survey study was conducted to determine the factors influencing the performance and emotional well-being of MNCPs during the COVID-19 pandemic. Participants invited to participate included doctors, midwives, nurses, sonographers, allied health staff (including cultural support workers, physiotherapists, social workers and dietitians), and administrative staff working in maternity or neonatal services. Eligible participants included permanent full-time, part-time, casual, and trainee staff (e.g., junior doctors or midwifery trainees) who were actively working in these services during the COVID-19 pandemic. Staff who were not actively working in maternity or neonatal services during the study period were excluded.

The questionnaire was developed specifically for this study, and the complete English-language version is provided in Supplementary File 1. This study adopted a pragmatic census approach, whereby all eligible maternity and neonatal staff across the participating hospitals were invited to participate. As the aim was to capture the experiences of the entire available workforce rather than to estimate population parameters from a sampled subset, no formal sample size calculation was performed. The final sample size was therefore determined by the total workforce size at the participating sites and the survey response rate during the recruitment period. Recruitment took place between October 2020 and March 2021.

### Sampling

Three Australian hospitals providing obstetric and neonatal care were invited to participate in the study, and all agreed to facilitate staff recruitment for the survey. The hospitals included one metropolitan tertiary hospital in Brisbane and two regional hospitals in South-East Queensland. Collectively, these hospitals have approximately 1,450 beds and facilitate around 8,500 births per year. Survey responses were collected using the secure online engagement platform Citizen Space [21].

The survey was distributed to employees by senior members from each hospital via email, including an explanation of the study and a link to the online questionnaire. To maximise participation across roles and rosters, recruitment included repeated email reminders, in-person promotion at varying times of day, and multiple completion modes (online, tablet-assisted, and paper).

Throughout recruitment, research nurses promoted survey uptake by distributing surveys directly to employees in person via tablets and printed copies. Printed surveys were also placed in staff common areas for later completion, and a locked container was provided at each site for secure submission of completed surveys. Research nurses subsequently transcribed all completed paper surveys into the online Citizen Space platform to allow integration with electronically submitted responses.

To reduce potential bias associated with self-reported information, neutral and non-leading language was used for all survey questions. Recall bias was minimised by administering the survey during a period of peak COVID-19 related service disruption in South-East Queensland.

Survey responses were collected anonymously, and no identifying personal information was recorded. Data were stored on secure password-protected servers accessible only to authorised members of the research team. Following data export, datasets were stored on secure Queensland Health network drives with restricted access.

### Data analysis

Data analysis was conducted using the Statistical Package for the Social Sciences (SPSS) software package version 29 (IBM Corp., Armonk, NY, USA). Descriptive statistics were used to summarise the sample characteristics. Exploratory factor analysis was conducted to develop a subscale representing staff emotional well-being, which served as the dependent variable in subsequent analyses. Additionally, exploratory factor analysis also generated subscales relating to lifestyle and workplace factors, which were used as independent variables in the analyses. These subscales were constructed from survey questions that captured the experiences of healthcare workers.

Pearson correlations were performed between continuous variables, while the point-biserial correlation was used to compare continuous variables with binary variables. Multiple linear regression analyses were performed to investigate the relationships between the identified dependent variable subscales and staff emotional well-being. Multiple hierarchical regression analyses were performed to investigate predictors of emotional well-being. Demographic and health-related variables (including age, First Nations status, sum of health conditions, and self-rated physical health) were entered in the first step of the model as control variables. Lifestyle factors were subsequently added in the second step, followed by substance use in the third step and workplace-related factors in the final step to assess their independent contributions to emotional well-being.

No subgroup analyses stratified by participant characteristics were performed, as the primary aim of the study was to identify predictors of emotional well-being across the overall sample.

## Results

Of the 517 respondents, eight discontinued the questionnaire prior to completion, leaving 509 completed questionnaires (98.4% completion rate), with no missing values. Valid survey responses were collected from 509/1064 eligible employees working across the sampling sites (response rate 47.8%). All responses included in the final analysis had complete data for variables used in factor and regression analyses. The total number of eligible employees was determined by consulting business managers and department heads at each hospital and counting full-time, part-time and casual staff in roles associated with maternal or neonatal care, including allied health and administrative positions.

### Demographics

Participant demographics for the study, including those used in the hierarchical regression model, are presented in Table 1. A total of 47.8% (509/1064) of participants responded to the survey. The majority were women with an average age of 37.2 (SD = 12.6). Midwives and nurses were the most represented healthcare staff in the study. The number of respondents who reported having one or more chronic health conditions was 40% (196/509), with a mean of 0.6 chronic conditions per person (SD = 0.83). Of the total respondents, 2% (10/509) identified as either Aboriginal or Torres Strait Islander, while men constituted only 4% (20/509).

**Table 1:** Demographic and employment characteristics of surveyed staff

Characteristic	Number	Percent (%)
**Total respondents**	509	100
**Gender**		
Male	20	4.0
Female	479	95.0
Other	5	1.0
**First Nations status**		
Either Aboriginal or Torres Strait Islander	10	2.0
Neither Aboriginal nor Torres Strait Islander	499	98.0
**Age**		
18–30	89	17.5
31–40	116	22.8
41–50	126	24.8
51–60	137	26.9
61+	37	7.3
**Physical health assessment**		
No diagnosed health conditions	302	60.6
One or more diagnosed health conditions	196	39.4
Would self-describe as being in good physical health	398	77.6
**Dependents**		
Provide care for school-aged dependents	242	47.5
Provide care for elderly dependents	74	14.5
**Occupation Role**		
Doctor	52	10.4
Midwife/ Nurse	378	75.9
Sonographer/ Radiographer	8	1.6
Administrative officer	42	8.4
Allied Health	18	3.6
**Work from home**		
Worked from home in any capacity during the pandemic	43	8.4

### Study measures developed through exploratory factor analysis

#### Emotional well-being

EFA of survey responses revealed a subscale that encapsulated staff emotional well-being (Table 2). Overall, a 7-item scoring system was extracted that measured a range of emotional states, including anxiety, depression, fatigue, distractibility, anger, isolation, and intrusive thoughts. Items were rated on a 5-point Likert scale ranging from ‘strongly disagree’ to ‘strongly agree.’ All seven items loaded strongly (loading > 0.6) onto the factor, indicating that they shared a common underlying dimension of emotional well-being [22]. The internal consistency of the scale was good (Cronbach’s alpha = 0.861) [22]. These results suggest that the seven items can be used as a reliable and valid measure of emotional well-being.

**Table 2:** Subscale measures and additional lifestyle factors used for multiple hierarchical analysis

Subscale	Survey Question	Score (Mean ± SD)	% Responded Agree or Strongly Agree
Emotional well-being	Felt anxious, worried, or overwhelmed during the peak of COVID-19	3.60 (1.10)	66.2%
	Felt overly fatigued or tired during the peak of COVID-19	3.49 (1.14)	59.2%
	Were unable to concentrate or focus during the peak of COVID-19	3.16 (1.08)	43.6%
	Felt sad, depressed, or unhappy during the peak of COVID-19	2.97 (1.15)	37.1%
	Felt lonely or isolated during the peak of COVID-19	2.80 (1.13)	33.6%
	Were irritable or easily angered during the peak of COVID-19	2.77 (1.10)	28.7%
	Had any negative thoughts or flashbacks about unpleasant experiences	2.13 (1.06)	12.3%
	**Aggregate Score (emotional well-being)**	2.96 (0.83)	-
Organisational response factors	Workplace carried out a timely and effective response to COVID-19 challenges	4.05 (0.72)	87.4%
	Workplace effectively communicated updated guidelines and protocols	4.01 (0.77)	84.5%
	Workplace undertook effective and timely measures to minimise exposure or risk of contracting COVID-19	3.97 (0.81)	81.7%
	Workplace provided sufficient training in the use of personal protective equipment (PPE) regarding COVID-19 preparedness	3.66 (0.94)	65.0%
	**Aggregate Score (Organisational Response Factors)**	3.92 (0.64)	-
Impact of COVID-19 in the workplace	Working at the hospital during COVID-19 made you more concerned for your health or increased your risk of contracting COVID-19	3.36 (1.09)	55.4%
	Patient outcomes have been negatively affected by the COVID-19 pandemic	3.31 (1.12)	50.1%
	Your ability to perform your duties was impaired by the COVID-19 pandemic	3.11 (1.12)	43.6%
	**Aggregate Score (Impact of COVID in Workplace)**	3.26 (0.81)	-
Substance use	Increased consumption of alcohol during the peak of COVID-19	2.4 (1.39)	31.8%
	Increased use of sleeping tablets or other sleep-assisting medications during COVID-19	1.64 (1.06)	11.0%
	Increased use of cigarettes or traditional tobacco products during COVID-19	1.35 (0.82)	4.4%
	Increased use of vaping products during COVID-19	1.25 (0.69)	2.4%
	Increased use of recreational drugs during COVID-19	1.23 (0.63)	1.8%
	**Aggregate Score (Substance use)**	1.57 (0.65)	-
Additional lifestyle factors	Average time spent per day watching or engaging with electronic media during the peak of COVID-19	2.88 (1.26)	-
	Average time engaging in structured physical exercise during the peak of COVID-19	2.34 (1.10)	-
	Average recreation time spent outdoors during the peak of COVID-19	2.04 (0.96)	-
	Average sleep at night during the peak of COVID-19	1.98 (0.65)	-

#### Organisational response factors

EFA identified a four-item subscale of protective organisational response factors that were hypothesised to affect staff well-being (Table 2). Items were rated on a 5-point Likert scale ranging from strongly disagree to strongly agree. All four items loaded strongly (loading > 0.7) onto the factor [22], and demonstrated good internal consistency (Cronbach’s alpha = 0.802) [22]. These results suggest that these four items can be used as a reliable and valid measure of workplace response to the pandemic.

#### Impact of COVID-19 on the workplace

A subscale representing the perceived impact of COVID-19 on the workplace was developed using exploratory factor analysis (EFA), comprising three items rated on a 5-point Likert scale (Table 2). All items loaded strongly onto a single factor (factor loading > 0.4), consistent with accepted thresholds for factor retention (14). Internal consistency was acceptable (Cronbach’s alpha = 0.669), meeting the minimum criterion of consistency of scales with fewer items with adequate factor loading (14). These results support the use of the three-item subscale as a valid and reliable measure of workplace impact.

#### Substance use

Maladaptive lifestyle behaviors involving substance use were also identified as a subscale through EFA and comprised of 5 items (Table 2). Items were rated according to whether their consumption of these substances had increased during the pandemic on a 5-point Likert scale ranging from ‘strongly disagree’ to ‘strongly agree.’ All five items loaded strongly (loading > 0.4) onto the factor [22], with good internal consistency (Cronbach’s alpha = 0.710) [22]. These results suggest that these five items can be used as a reliable and valid measure of substance-related maladaptive behaviors.

#### Additional lifestyle factors

In addition to the substance use factors that were hypothesised to negatively impact well-being, other lifestyle variables were assessed that were thought to convey a beneficial effect on staff well-being (Table 2). As responses to these questions lacked a unified scoring framework, EFA could not be applied to identify underlying patterns or dimensions; therefore, their effects on staff well-being were analysed individually as separate variables.

### Impact on emotional well-being

#### Correlation of personal and workplace factors with employee emotional well-being

During the peak of the COVID-19 pandemic, the majority of the sampled MNCP workforce reported feelings of anxiety and fatigue (Table 2). No significant correlation was found between emotional well-being and participants’ care for school-aged dependents, care of elderly dependents, gender, or First Nations status (Table 3). Older age was significantly (*p* < 0.05) associated with increased emotional well-being (Table 3). A positive self-assessment of health was significantly (*p* < 0.01) associated with increased emotional well-being (Table 3). A greater number of diagnosed health conditions and a higher perceived risk of contracting COVID-19 in the workplace were significantly (*p* < 0.01) associated with reduced emotional well-being.

**Table 3:** Correlations between emotional well-being, demographics, self-rated health, and perceived workplace COVID-19 risk among staff

Variable		1	2	3	4	5	6	7	8	9
1	Emotional well-being	1.00								
2	Elderly dependents	-0.041	1.00							
3	Young dependents	-0.141	-0.095	1.00						
4	Gender	-0.032	0.081	-0.065	1.00					
5	First Nations status	-0.081	0.022	0.135	0.019	1.00				
6	Age	0.110 *	0.180 **	-0.175	-0.085	-0.023	1.00			
7	Sum of health conditions	-0.159 **	0.081	-0.136	0.027	0.092 *	0.226 **	1.00		
8	Self-describes as being in good health	0.164 **	-0.013	0.344 **	-0.111 *	-0.053	-0.010	-0.252 **	1.00	
9	Assessment of workplace risk	-0.306 **	0.017	-0.039	0.052	-0.008	-0.031	0.046	-0.065	1.00

*Correlation is significant at the 0.05 level (2-tailed)

**Correlation is significant at the 0.01 level (2-tailed)

Lifestyle factors hypothesised to be protective (adequate sleep, exercise, outdoor time, and avoidance of excessive media) were found to be significantly correlated with emotional well-being. A significant correlation was also demonstrated between lower levels of emotional well-being and increased substance use (alcohol, sleeping pills, smoking, vaping and recreational drug use) (See Table 4).

**Table 4:** Multiple hierarchical regression model predicting maternity and neonatal care workers’ emotional well-being

Hierarchical analysis level	β	95% CI for β	t	Sig	∆*R*^2^	Cumulative *R*^2^
**Step 1**					**0.66**	**0.066**
First Nations status	− 0.339	− 0.855, 0.178	-1.288	0.198		
Age	0.096	0.038, 0.154	3.252	0.001		
Sum of health conditions	− 0.157	− 0.249, − 0.065	-3.355	< 0.001		
Perceived physical health	0.116	0.033, 0.199	2.732	0.007		
**Step 2**					**0.132**	**0.198**
First Nations status	-0.274	− 0.756, 0.208	-1.116	0.265		
Age	0.101	0.045, 0.158	3.516	< 0.001		
Sum of health conditions	− 0.109	− 0.195, − 0.022	-2.463	0.014		
Perceived physical health	0.047	− 0.035, 0.129	1.134	0.257		
Sleep	0.319	0.213, 0.424	5.932	< 0.001		
Exercise	0.011	− 0.058, 0.080	0.316	0.752		
Time outdoors	0.172	0.097, 0.248	4.487	< 0.001		
Avoidance of media	0.092	0.037, 0.147	3.275	0.001		
**Step 3**					**0.035**	**0.232**
First Nations status	− 0.152	− 0.627, 0.323	− 0.628	0.531		
Age	0.089	0.033, 0.144	3.139	0.002		
Sum of health conditions	− 0.086	− 0.171, − 0.001	-1.981	0.048		
Perceived physical health	0.057	− 0.023, 0.137	1.392	0.165		
Sleep	0.278	0.173, 0.383	5.211	< 0.001		
Exercise	0.003	− 0.065, 0.071	0.097	0.923		
Time outdoors	0.166	0.092, 0.240	4.412	< 0.001		
Avoidance of media	0.076	0.021, 0.130	2.740	0.006		
Substance use	− 0.249	− 0.354, − 0.145	-4.691	< 0.001		
**Step 4**					**0.119**	**0.351**
First Nations status	0.021	− 0.418, 0.460	0.095	0.925		
Age	0.061	0.009, 0.112	2.311	0.021		
Sum of health conditions	− 0.093	− 0.172, − 0.014	-2.318	0.021		
Perceived physical health	0.027	− 0.047, 0.101	0.710	0.478		
Sleep	0.230	0.133, 0.327	4.640	< 0.001		
Exercise	0.031	− 0.032, 0.094	0.974	0.330		
Time outdoors	0.130	0.061, 0.198	3.714	< 0.001		
Avoidance of media	0.064	0.014, 0.114	2.517	0.012		
Substance use	− 0.210	− 0.307, − 0.114	-4.282	< 0.001		
Organisational response factors	0.092	− 0.004, 0.189	1.886	0.060		
COVID-19 impact on workplace	− 0.345	− 0.422, − 0.268	-8.772	< 0.001		

Employees’ perceptions of COVID-19’s negative influence on the workplace (increased concern for personal health, impact on ability to perform usual duties, negative impact on patient care) were significantly associated with decreased emotional well-being (Table 4). Conversely, employees’ positive assessments of their hospital’s response to the organisational challenges of COVID-19 (timely response to challenges of COVID-19, effective communication, minimising infectious exposure, adequate PPE training) were significantly associated with greater emotional well-being (Table 4).

#### Multiple hierarchical regression analysis

Multiple hierarchical regression analyses were conducted to explore predictors of emotional well-being among MNCPs (Table 4). At hierarchical level one, demographics and health factors were assessed. ‘Age’, ‘sum of health conditions’, and ‘perceived physical health’ were found to be predictors of emotional well-being. At hierarchical level two, lifestyle factors were added to the predictive model. ‘Age’ and ‘sum of health conditions’ remained predictors, while additionally ‘sleep’, ‘time spent outdoors’, and ‘media avoidance’ were the new predictors. At hierarchical level three, ‘substance use’ patterns were included, which was found to be an additional predictor, while the previous predictors remained significant. At the fourth hierarchical level, ‘COVID-19 impact on the workplace’ and ‘organisational factors’ were assessed. The strongest predictors of emotional well-being were the COVID-19 associated impacts on workplace functioning, substance use, time spent outdoors and sleep. Overall, the final model with all predictors explained 35.1% of the variance in emotional well-being (Adjusted R² = 0.351, p < 0.001).

## Discussion

This study examined the impact of the COVID-19 pandemic on the maternity and neonatal workforce across three obstetric hospitals in South-East Queensland. In line with previous Australian research [9, 12, 20], our findings indicated that the majority of surveyed MNCPs reported substantial levels of anxiety and fatigue. A further aim was to identify potentially modifiable factors that could be targeted to support staff well-being and foster a more resilient workforce. To our knowledge, this is the first study to stratify the relative contributions of various workplace and personal factors to MNCP well-being—an approach that remains highly relevant in the post-pandemic context to inform future preparedness and response efforts.

### Workplace factors predicting staff well-being

Multiple hierarchical analyses identified the ‘Impact of COVID-19 on the Workplace’ subscale as the most significant contributor to our model of MNCP emotional well-being. This subscale amalgamates various workplace factors, including perceived risk of contracting COVID-19, staff ability to perform their duties, and adverse patient outcomes. As our model highlights the significant impact of these factors on staff well-being, we propose that these represent key targets for interventions aimed at enhancing employee capacity and well-being during future health crises.

Regarding the impact of staff’s perceived risk of contracting COVID-19, other studies have also demonstrated that staff stress and anxiety related to the risk of contracting COVID-19 not only involved personal health concerns but also extended to fears of infecting family members [23, 24]. Consistent with this finding, the proper provision and implementation of PPE has been shown to alleviate staff anxiety and mitigate traumatic experiences during the pandemic [24]. To help staff maintain performance during pandemic challenges, workplace strategies like peer support programs, staff briefings and debriefings, and regular e-bulletins have been shown to improve staff well-being [25].

Our study also aligns with the consensus that the COVID-19 pandemic negatively impacted maternity and neonatal staff by restricting woman-centred care [7, 9, 26]. A large international survey of health workers identified six areas where COVID-19 affected the provision of respectful maternity care: (i) restrictions on family involvement, (ii) reduced emotional support for women, (iii) reduced physical support for women, (iv) compromised standards of care, (v) increased rate of medically unnecessary caesarean sections, and (vi) overwhelmed healthcare staff responding to evolving guidelines and stricter infection control measures [7]. Considering these findings, service changes should be customised to address individual needs, avoiding blanket risk-reduction strategies while prioritising staff flexibility to accommodate necessary exceptions and minimise adverse impacts on patients and staff during major health crises.

### Personal factors predicting staff well-being

Beyond the workplace factors, three personal factors significantly contributed to our predictive model of staff well-being. Reported average sleep duration, increased substance use, and dedicated outdoor recreational time all significantly contributed to the prediction of MNCP emotional well-being in the multiple hierarchical analyses.

During the pandemic, a meta-analysis reported the prevalence of insomnia or sleep disturbances among healthcare workers at 39.45%, with a higher prevalence for nurses compared with doctors, as well as a higher prevalence among female staff and frontline healthcare workers [6]. Specific interventions to monitor and improve sleep quality among healthcare staff are likely to play a key role in improving staff well-being. Interventions such as online mindfulness-based stress reduction programs and meditation programs have been shown in clinical trials to significantly improve sleep quality for COVID-19 intensive care nurses [27] and in the general population [28]. As our study identified a correlation between poor sleep and staff well-being, alongside the reported prevalence of sleep disturbances during the pandemic [6], addressing the contributors to poor sleep should be prioritised to enhance staff health and resilience.

Substance use habits have been shown to be prevalent during the pandemic, with a meta-analysis demonstrating that healthcare workers increased their alcohol consumption and that around one-fifth of healthcare workers used alcohol to dangerous levels [29]. A qualitative study found that nurses increased their alcohol consumption during the pandemic, attributing the rise to heightened workload and stress, as well as staff viewing alcohol as a form of reward [30]. This is potentially a critical safety issue that healthcare services must carefully consider.

Our model’s finding that increased dedicated outdoor time predicted MNCP well-being is similar to other studies that have linked outdoor activity to better mental health outcomes [31, 32]. Hospitals could encourage this behavior by promoting outdoor activities and developing therapeutic gardens, which have demonstrated benefits for both staff and patient well-being [33].

Synthesising these findings, several practical pathways emerge for health service implementation. Key priorities include consistent leadership communication and debriefing structures, timely PPE training, protected breaks with access to restorative spaces, and strategies to address fatigue and unhealthy coping behaviors. While our study focused on maternity and neonatal services, these levers operate at the system level and are scalable across diverse healthcare settings, supporting staff resilience and sustaining service quality in future crises.

### Limitations

Our study included few male and First Nations respondents, reflecting the demographics of the MNCP workforce in South-East Queensland. Additionally, the absence of pre-pandemic data limits our ability to establish baseline comparisons regarding the pandemic’s impact on MNCP well-being. Participation in the survey was voluntary, introducing the possibility of self-selection bias. Individuals with stronger views or experiences related to workplace conditions during the pandemic may have been more motivated to participate. It is plausible that staff experiencing higher levels of stress or fatigue were more likely to respond. Conversely, it is also possible that more engaged staff, potentially those with stronger workplace support or adaptive coping strategies, may have been more likely to participate. Consequently, the findings may overestimate the magnitude of associations, although the direction of potential responder bias remains uncertain. Nevertheless, the relatively high response rate (47.8%) suggests substantial engagement from the workforce. Furthermore, our study only included the South-East Queensland workforce, potentially limiting its generalisability to other settings. Finally, our survey was conducted prior to widespread vaccination, which may have influenced staff perceptions of the threat of contracting COVID-19.

## Conclusion

The COVID-19 pandemic presented a unique opportunity to study the factors influencing staff well-being under extreme and unexpected pressures on healthcare delivery. This is the first study to examine the effect of the COVID-19 pandemic on the broader spectrum of hospital staff in the maternity and neonatal healthcare setting in Australia. Our model identifies the workplace-related and personal factors that most strongly influence MNCP well-being, providing valuable targets for initiatives aimed at enhancing staff well-being and performance. Based on the multiple hierarchical analyses, the most impactful factors to target for improving staff well-being are enhancing sleep quality, increasing time spent outdoors, reducing substance use, and minimising the workplace impact of COVID-19. These strategies should be embedded into long-term service planning, positioning staff well-being as a cornerstone of workforce retention, service quality, and patient safety. Future studies should investigate whether hospital setting modifies the association between workplace stressors and emotional well-being. Tertiary centres may experience higher acuity, referral complexity, and system coordination demands, whereas secondary settings may face resource limitations or staffing constraints. Examining these contextual differences using stratified or multilevel modelling approaches may clarify whether intervention priorities should differ by service environment.

## Supplementary Information

Below is the link to the electronic supplementary material.

Supplementary Material 1

## Data Availability

The datasets used and/or analysed during the current study are available from the corresponding author on reasonable request.

## Abbreviations

CI: Confidence interval
COVID-19: Coronavirus disease 2019
EFA: Exploratory factor analysis
HREC: Human Research Ethics Committee
MNCP: Maternity and neonatal care provider
PPE: Personal protective equipment
R²: Coefficient of determination
SD: Standard deviation
SPSS: Statistical Package for the Social Sciences
