# Supplementary material for: Workplace and personal predictors of well-being in the broader maternity and neonatal workforce during COVID-19: evidence from a multicentre survey
Source: BMC Health Serv Res. 2026 Jul 28;26:1468. doi: 10.1186/s12913-026-14588-x (PMC13700441; doi:10.1186/s12913-026-14588-x)
Supplement: Supplementary file 1 [file 12913_2026_14588_MOESM1_ESM.docx]

# **Supplemental file 1: Questionnaire for participants**

**Introduction:**

Thank you for participating in the following survey, which asks about your experience working in the maternity and neonatal health care setting during the novel coronavirus (COVID-19) pandemic. The information you provide will assist the Metro North Hospitals and Health service in improving our response to the challenges posed by COVID-19.

You are eligible to participate in this survey if you are a health care worker in a “Queensland Health Metro North” hospital (Royal Brisbane, Redcliffe and Caboolture Hospitals) and are working in maternity or neonatal health as either a: doctor, midwife, registered nurse, enrolled nurse, sonographer / radiographer, paramedic or administrative officer.

Throughout this survey Novel Coronavirus will be referred to as COVID-19.

***Please complete this survey only once.***

**Consent to Participate:**

Participation in this survey is completely voluntary and information provided by you will be anonymous. By completing the on-line survey, you are telling us that you:

- Understand what you have read, and
- Consent to take part in the survey

**Important information:**

If you feel at any time that being involved in this survey has contributed to any feelings of unease, anxiety or has negatively affected you in any way and you would like to talk with someone not involved in the study, please contact the “Benestar” hotline, or alternatively arrange an appointment to talk with a general practitioner (GP).

**There contact details are:**

In Australia: [1300 360 364](tel:1300-360-364)

New Zealand: [0800 360 364](tel:0800-360-364)

Internationally: [+61 2 8295 2292](tel:+61-2-8295-2292)

“Benestar” are an organisation that works with Queensland health to provide free support for Queensland health employees. They can be contacted over the phone, free of charge, 365 days a year, 24/7 to provide support and counselling regarding any personal wellbeing concerns: physical, mental, social or financial.

If you would like more information about this project, please contact Dr Alexander Lindgren or Dr Alka Kothari through the Redcliffe Hospital switchboard (3883 7777). If you have concerns about how this study is conducted please contact the Human Research Ethics Committee 1300 064 672 in Australia, or +61 2 6217 9000 for international callers, or email nhmrc@nhmrc.gov.au.

**Thank you for your participation in this survey**

**Impact of the COVID-19 pandemic on Maternity health care providers**

**PERSONAL PHYSICAL HEALTH IMPACTS OF COVID-19:**

1. Have you been diagnosed with COVID-19?
   a. Yes
   b. No
2. Have you been tested for COVID-19?
   a. Yes
   b. No
3. Have you been worried you may have it, but have not been tested?
   a. Yes
   b. No
4. Have been diagnosed with a health condition that places you at increased risk of COVID-19 – select all that apply:
   a. Asthma
   b. Chronic kidney disease being treated with dialysis
   c. Chronic lung disease
   d. Diabetes
   e. Haemoglobin or red blood cell disorders
   f. Immunocompromised
   g. Liver disease
   h. People aged 65 years and older
   i. Serious heart conditions
   j. Obesity
   k. Other: (Please specify) _____________
5. Would you describe yourself as being in good physical health?
   a. Strongly Disagree
   b. Disagree
   c. Neutral
   d. Agree
   e. Strongly Agree
6. Would you say that working at the hospital in maternity health care during the COVID-19 pandemic has ***made you more concerned for your health or has placed you at a greater risk of exposure to contracting COVID-19? BAD***
   a. Strongly Disagree
   b. Disagree
   c. Neutral
   d. Agree
   e. Strongly Agree
7. Would you say that the hospital where you work has ***undertaken effective and timely measures to minimise your exposure to or risk of contracting COVID-19?***
   a. Strongly Disagree
   b. Disagree
   c. Neutral
   d. Agree
   e. Strongly Agree
8. In your capacity as health care worker setting how would you describe your exposure to patients ***(please select the option that applies the most to your usual role).***
   a. ***PROLONGED CLOSE PHYSICAL CONTACT*** (e.g. working in birth suite, maternity ward, performing regular physical examinations, or regularly taking vital signs etc).
   b. ***LIMITED CLOSE PHYSICAL CONTACT*** (e.g. either frequently speaking face to face with or only occasionally throughout the day physically touching patients).
   c. ***MINIMAL CONTACT WITH PATIENTS*** (e.g. only occasionally speaking face to face with patients or working in areas where patients are present but not directly touching patients as part of your usual duties).
   d. ***NO SIGNIFICANT PHYSICAL CONTACT*** (e.g. working in a setting that is removed from patient areas).

**WORK PROCEDURAL AND PATIENT OUTCOMES OF COVID-19:**

9. Do you feel that your workplace has carried out a timely and effective response to the challenges of COVID-19?
   a. Strongly Disagree
   b. Disagree
   c. Neutral
   d. Agree
   e. Strongly Agree
10. Would you say that your workplace has effectively communicated to you with updated guidelines and protocols to assist you in your work duties?
    a. Strongly Disagree
    b. Disagree
    c. Neutral
    d. Agree
    e. Strongly Agree
11. Would you say that your workplace has engaged with you in sufficient theoretical and practical training in the use of personal protective equipment (PPE) with regards to COVID-19 preparedness?
    a. Strongly Disagree
    b. Disagree
    c. Neutral
    d. Agree
    e. Strongly Agree
12. Would you say that your ability to effectively perform your usual duties has been impaired by the COVID-19 pandemic?
    a. Strongly Disagree
    b. Disagree
    c. Neutral
    d. Agree
    e. Strongly Agree
13. “Would you say that patient outcomes have been negatively affected by the COVID-19 pandemic?”
    a. Strongly Disagree
    b. Disagree
    c. Neutral
    d. Agree
    e. Strongly Agree
14. Please tell us if you have observed any positive or effective ***strategies or protocols*** at your workplace that have benefited staff or patients during the COVID-19 pandemic
    a. Free text…
15. Please tell us if you have observed any negative or unhelpful ***strategies or protocols*** at your workplace that have affected staff or patients during the COVID-19 pandemic
    a. Free text…

**SOCIAL IMPACTS OF COVID-19:**

16. Are you still currently employed in the Queensland Health Metro North Health Service?
    a. Yes
    b. No
17. Have you taken either paid or unpaid leave during the COVID-19 pandemic?
    a. Yes – for one month or more
    b. Yes – between 1 to 4 weeks
    c. Yes – for less than 7 days
    d. No
18. Have you been working from home in any capacity during the COVID-19 pandemic?
    a. Yes – for one month or more
    b. Yes – between 1 to 4 weeks
    c. Yes – for less than 7 days
    d. No
19. Do you have elderly dependant/s that you provide care for?
    a. Yes
    b. No (if no skip to question 22.)
20. Regarding your elderly dependant/s do they:
    a. Live at home with you
    b. Live in a household separate from your usual residence
    c. Live in an aged care facility
21. Would you say that providing care for your Elderly dependant/s has led to increased stress or difficulties in your household
    a. Strongly Disagree
    b. Disagree
    c. Neutral
    d. Agree
    e. Strongly Agree
22. Do you have children or young dependant/s that you provide care for?
    a. Yes
    b. No (if no skip to question 26.)
23. Do your children or young dependant/s normally (pre COVID-19) attend school or childcare?
    a. Yes
    b. No (if no skip to question 26.)
24. Have any of your children or young dependant/s been engaged in ***home-based care / home schooling / online classes*** because of the COVID-19 pandemic?
    a. Yes
    b. No (if no skip to question 26.)
25. ***Home-based care / home schooling / online classes*** has led to increased stress or difficulties in your household
    a. Strongly Disagree
    b. Disagree
    c. Neutral
    d. Agree
    e. Strongly Agree

**EMOTIONAL AND MENTAL WELLBEING:**

26. Would you say that ***during the peak of COVID-19*** you were:

** for each question a) strongly disagree, b) disagree, c) neutral, d) agree, e) strongly agree**

a. Felt anxious, worries or overwhelmed?
b. Felt sad, depressed, or unhappy?
c. Been able to enjoy life?
d. Felt overly fatigued or tired?
e. Been able to concentrate or focus?
f. Been irritable or easily angered?
g. Felt lonely or isolated?
h. Had any negative thoughts or flashbacks about unpleasant experiences?

**LIFESTLYE INFLUENCES / COPING STRATEGIES:**

*****The following questions: 27-30 apply to the *during the peak of COVID-19* *****

27. On an average, how many hours did you sleep at night?
    a. 5 or fewer hours
    b. 6-7 hours
    c. 8-9 hours
    d. >9 hours
28. How many days per week did you engage in:
    a. ***Structured physical exercise*** (e.g. walking / running / sports etc.)
       i. None
       ii. 1-2 days
       iii. 3-4 days
       iv. 5-6 days
       v. Daily
    b. How many days per week did you spend ***dedicated recreational time outdoors*** (e.g. picnics / play with family / friends / pets)
       i. None
       ii. 1-2 days
       iii. 3-4 days
       iv. 5-6 days
       v. Daily
29. How much time did you spend ***on average per day*** watching or engaging with television / online video platforms / social media / recreational web browsing playing video games?
    a. Less than 1 hour
    b. 1-2 hours
    c. 2-3 hours
    d. 3-4 hours
    e. 4+ hours
30. Would you say that ***during the peak of COVID-19*** you had increased your consumption of:

** for each question a) strongly disagree, b) disagree, c) neutral, d) agree, e) strongly agree**

a. “Sleeping tablets” or other sleep assisting prescription or over the counter medicines
b. Alcohol
c. Cigarettes or traditional tobacco products
d. Vaping or electronic nicotine devices
e. Recreational drugs

**DEMOGRAPHIC QUESTIONS:**

31. Where do you work?
    a. Royal Brisbane Women’s Hospital
    b. Redcliffe Hospital
    c. Caboolture Hospital
32. What is your role?
    a. Doctor
       i. Specialist
       ii. Registrar
       iii. Resident
       iv. Intern
    b. Midwife / Nurse
       v. Registered midwife
       vi. Graduate midwife
       vii. Registered Nurse
       viii. Enrolled Nurse
    c. Sonographer / Radiographer
    d. Paramedic / Queensland Ambulance Service clinical worker
    e. Administrative Officer
33. What is your age:
    a. 18-20
    b. 21-30
    c. 31-40
    d. 41-50
    e. 51-60
    f. 61 or above
34. What is your gender?
    a. Female
    b. Male
    c. Rather not say
35. Do you identify as Aboriginal or Torres Strait Islander?
    a. Yes
    b. No
36. Where do you work in the hospital (Tick as many as appropriate)?
    a. Birth Suite
    b. Maternity Ward
    c. Antenatal clinic
    d. Theatre
    e. Retrievals: NeoRESQ / Q.A.S
    f. Medical Imaging
    g. Other: (Please specify) _____________
37. How long have you been working in your area of speciality? (include your training years)
    a. Less than 5 years
    b. 5-10 years
    c. 11-20 years
    d. 20-30 years
    e. 31 years or over
38. In the last 12 months, in what capacity were you predominately working in the Maternity health care setting?
    a. Full time
    b. Part time

**Important information:**

If you feel at any time that being involved in this survey has contributed to any feelings of unease, anxiety or has negatively affected you in any way and you would like to talk with someone not involved in the study, please contact the “Benestar” hotline, or alternatively arrange an appointment to talk with a general practitioner (GP).

**There contact details are:**

In Australia: [1300 360 364](tel:1300-360-364)

New Zealand: [0800 360 364](tel:0800-360-364)

Internationally: [+61 2 8295 2292](tel:+61-2-8295-2292)

“Benestar” are an organisation that works with Queensland health to provide free support for Queensland health employees. They can be contacted over the phone, free of charge, 365 days a year, 24/7 to provide support and counselling regarding any personal wellbeing concerns: physical, mental, social or financial.
